# Resistance of *Klebsiella pneumoniae* to Phage hvKpP3 Due to High-Molecular Weight Lipopolysaccharide Synthesis Failure

**DOI:** 10.1128/spectrum.04384-22

**Published:** 2023-04-06

**Authors:** Huaixin Geng, Lingjie Song, Xianggui Yang, Siyu Xing, Rui Wang, Ying Xu, Xu Jia, Guangxin Luan

**Affiliations:** a Non-coding RNA and Drug Discovery Key Laboratory of Sichuan Province, Chengdu Medical College, Chengdu, Sichuan, China; b Department of Laboratory Medicine, Clinical Medical College and the First Affiliated Hospital of Chengdu Medical College, Chengdu, Sichuan, China; University of California, San Diego

**Keywords:** hypervirulent *Klebsiella pneumoniae*, phage resistance, lipopolysaccharide, glycosyltransferase

## Abstract

The spread of multidrug resistant and hypervirulent Klebsiella pneumoniae has recently increased. Phages have been considered alternatives for treating infections caused by tenacious pathogens. Our study describes a novel lytic Klebsiella phage, hvKpP3, and we obtained spontaneous mutants, hvKpP3R and hvKpP3R15, of hvKpLS8 strain that showing strong resistance to the lytic phage hvKpP3. Sequencing analysis showed that nucleotide-deletion mutations of the glycosyltransferase gene (*GT*) and *wcaJ* genes, located in the lipopolysaccharide (LPS) gene cluster and the capsular polysaccharide (CPS) gene cluster, respectively, led to phage resistance. The *wcaJ* mutation confers the inhibition of phage adsorption by affecting the synthesis of hvKpP3R15 capsular polysaccharide, indicating that the capsule is the main adsorption receptor for bacteriophage hvKpP3. Interestingly, the phage-resistant mutant hvKpP3R has a loss-of-function mutation in *GT,* which is responsible for lipopolysaccharide biosynthesis. This results in the loss of high-molecular weight lipopolysaccharide (HMW-LPS), and alteration of the lipopolysaccharide structure of the bacterial cell wall confers resistance to phages. In conclusion, our study provides a detailed description of phage hvKpP3 and provides new insights into phage resistance in K. pneumoniae.

**IMPORTANCE** Multidrug-resistant (MDR) Klebsiella pneumoniae strains pose a particular threat to human health. Therefore, it is very important for us to isolate phage and overcome phage resistance. In this study, we isolated a novel phage belonging to the *Myoviridae* family, hvKpP3, that exhibited high lytic activity against K2 hypervirulent K. pneumoniae. We demonstrated the excellent stability of phage hvKpP3 through *in vitro* and *in vivo* experiments, indicating its potential as a candidate for future clinical phage therapy. Furthermore, we identified that loss of function in the glycotransferase gene (*GT*) caused the failure of HMW-LPS synthesis, leading to phage resistance, which provides new insights into phage resistance in K. pneumoniae.

## INTRODUCTION

Klebsiella pneumoniae is an opportunistic pathogen that is widely found in the natural environment (1). K. pneumoniae produces at least 79 chemically diverse capsule serotypes, with the K2 serotype being one of the most frequently isolated serotypes in hypervirulent K. pneumoniae epidemic strains (2). Most complications of liver abscesses, endophthalmitis, and meningitis are caused by the hypervirulent K. pneumoniae K2 serotype (3). Currently, K. pneumoniae is included in the World Health Organization’s list of multidrug-resistant (MDR) bacteria and has become one of the most challenging antibiotic-resistant pathogens (4, 5). Given the increasing number of multidrug-resistant and hypervirulent K. pneumoniae cases, phages have been considered as a therapeutic approach (6).

A large number of Klebsiella bacteriophages have been isolated and characterized, and the GenBank database currently includes more than 120 genomes of Klebsiella phages (7). However, only a few phages have been described which specifically act against K. pneumoniae strains with the K2 serotype (8–10). Furthermore, the inevitability of phage resistance is a challenge in clinical treatment. Resistance can arise by preventing phage adsorption and DNA entry, cutting phage nucleic acids, and abortive infection systems. Mutations affecting phage receptors, including capsular polysaccharides and lipopolysaccharides, are the most frequent causes of phage resistance (11). Lipopolysaccharides (LPS) are composed of three covalently bound components: lipid A, an R-core oligosaccharide, and an O-antigen. LPS play an important role in bacterial evasion of host cell defenses and are correlated with many pathogenic properties (12). A previous study showed that the glycosyltransferase gene (*GT*) is responsible for the biosynthesis of the O-antigen of bacterial lipopolysaccharides (13). However, little is known about the mechanisms underlying *GT*-mediated resistance induced by the host to avoid invasion by phages. Therefore, we conducted the following investigation to provide new approaches for overcoming phage resistance in bacteria.

In this study, we isolated a novel bacteriophage, hvKpP3, capable of lysing the K2 serotype hypervirulent K. pneumoniae strain, from a wastewater treatment plant in Chengdu, Sichuan. We determined the general biological properties of hvKpP3, analyzed its genomic characteristics, and established a Galleria mellonella infection model to demonstrate its therapeutic efficacy, providing a scientific basis for the future application of K. pneumoniae phages in clinical treatment. Furthermore, sequence analysis showed that the *GT* gene had a mutation. This gene’s role in the phage-resistant mutant hvKpP3R was comprehensively investigated.

## RESULTS

### Phage isolation, morphology, and host range.

**FIG 1:**
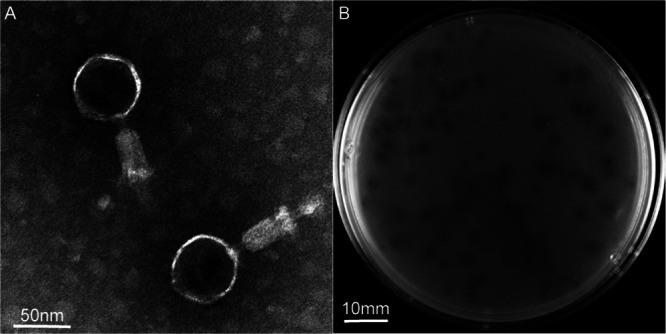
Structural characterization and plaque morphology of isolated phages. (A) Transmission electron micrograph (TEM) of phage hvKpP3. Scale bar = 50 nm. (B) Plaques of phage hvKpP3 on Klebsiella pneumoniae hvKpLS8. Scale bar = 10 mm.

The clinical strain hvKpLS8 was characterized as an hvKP by nine virulence-associated genes: *ureA*, *wabG*, *fimH*, *entB*, *ycf*, *ybtS*, *iutA*, *aerobact*, and *uge*. This clinical strain belongs to the K2 capsular serotype. Using K. pneumoniae hvKpLS8 as an indicator strain, we isolated a previously unidentified phage and designated it Klebsiella phage vB_KpnM_cmc20193 (referred to as hvKpP3). After further isolation and purification, the titer of hvKpP3 was 3.12 × 10^10^ PFU/mL. Transmission electron microscopy (TEM) showed that the phage had a typical icosahedral structure and a contractile tail, with a head diameter of approximately 58 ± 1 nm and tail length of approximately 91 ± 2 nm, which are characteristic features of phages in the family *Myoviridae* (Fig. 1A). In addition, the phage formed 1- to 2-mm diameter individual plaques on its host bacterial lawn (Fig. 1B). Relatively large (4- to 6-mm diameter) turbid rings (halo zones) were observed around the clear plaques after incubation. This may be a result of the differential migration of diffused soluble depolymerases (10, 14, 15). Halos expanded over time, whereas plaques remained the same size. Notably, host range experiments also confirmed that only strains belonging to the K2 capsular serotype were specifically targeted by this phage. Lysis did not occur in the other strains tested (K57, K64, and K84) (Table 1). Phages targeting K. pneumoniae capsular-type K2 strains showed specific host ranges, indicating that they may be useful for K. pneumoniae typing.

**TABLE 1:** Host range of phages

Strain	K type	hvKpP3	Source
hvKpLS8	K2	√^*a*^	Sixth Affiliated Hospital of Wenzhou Medical University
hvKpLS7	K57	×	Sixth Affiliated Hospital of Wenzhou Medical University
355	K64	×	General Hospital of Western Theater Command of PLA
356	K64	×	General Hospital of Western Theater Command of PLA
KPn396	K2	√	Shanghai Renji Hospital
KPn1077	K2	√	Shanghai Renji Hospital
KPn1953	K2	√	Shanghai Renji Hospital
KPn1278	K2	√	Shanghai Renji Hospital
KP0734	K2	√	Shanghai Renji Hospital
KP2115	K2	√	Shanghai Renji Hospital
KP1072	K2	√	Shanghai Renji Hospital
377	K84	×	General Hospital of Western Theater Command of PLA

a “√” as clear lysis; “×” as no lysis.

### Phage characterization.

**FIG 2:**
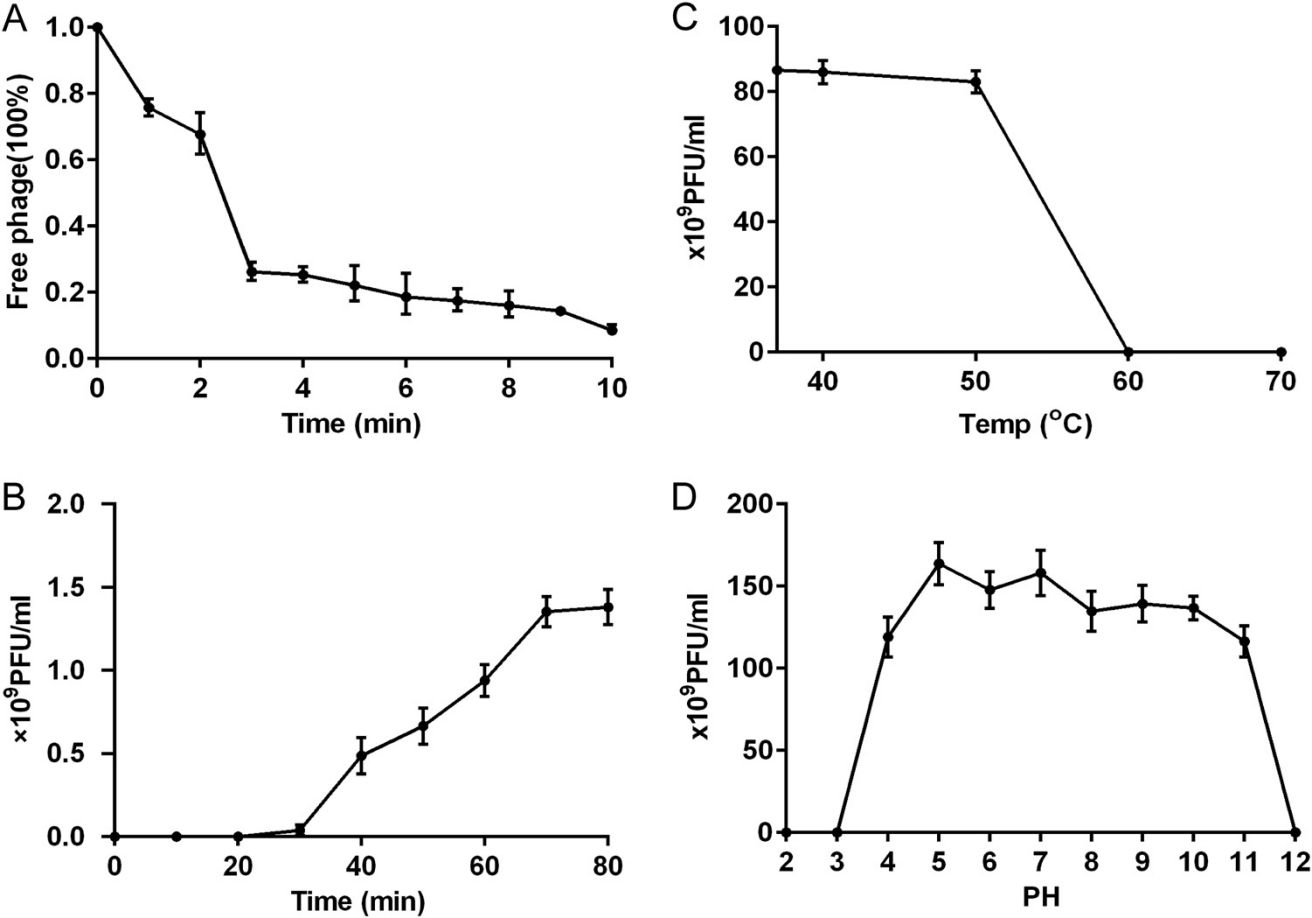
Characterization with K. pneumoniae phage hvKpP3. (A) Adsorption of the phage hvKpP3. (B) One-step growth curve of phage hvKpP3. (C) Tolerance of phage hvKpP3 to different pH. (D) Tolerance of phage hvKpP3 to different temperatures.

The phage adsorption assay showed that more than 90% of phage particles were adsorbed onto the host cell within 10 min (Fig. 2A). A one-step growth experiment was performed to determine the latent period and burst size of the phage on the host. The one-step growth curve of the phage showed an incubation period of approximately 30 min (Fig. 2B). During this period, the number of plaques did not increase, indicating that the phage had not completed its replication and assembly. After reaching the plateau phase at 60 min, the burst size was approximately 94 PFU/cell, indicating that the phage maintained high replication capacity and lytic activity. The physical tolerance of a phage is also critical for its application under different conditions. Phages were stable in the pH range of 4 to 11 and at 60°C (Fig. 2C and D). These results are comparable to the stability of phages isolated in previous studies (5).

The efficacy of phage therapy was evaluated *in vivo* using a G. mellonella larvae model. The survival rate of larvae infected with the host strain hvKpLS8 was only 15%. In the phage-treated group, the survival rate was significantly higher, with a 3-day survival rate of 65% (Fig. S1). Furthermore, injection of either phosphate-buffered saline (PBS) or phage resulted in a high survival rate, demonstrating the safety of phages *in vivo*. These data indicate that the phage has promising potential for the treatment of infections.

### Whole-genome characterization and analysis.

**FIG 3:**
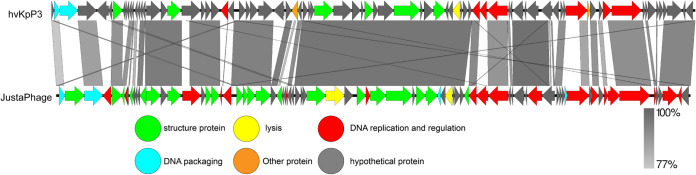
Phylogenetic relations of K. pneumoniae phage hvKpP3 based on the whole-genome sequence generated by Mega 6.0.

**FIG 4:**
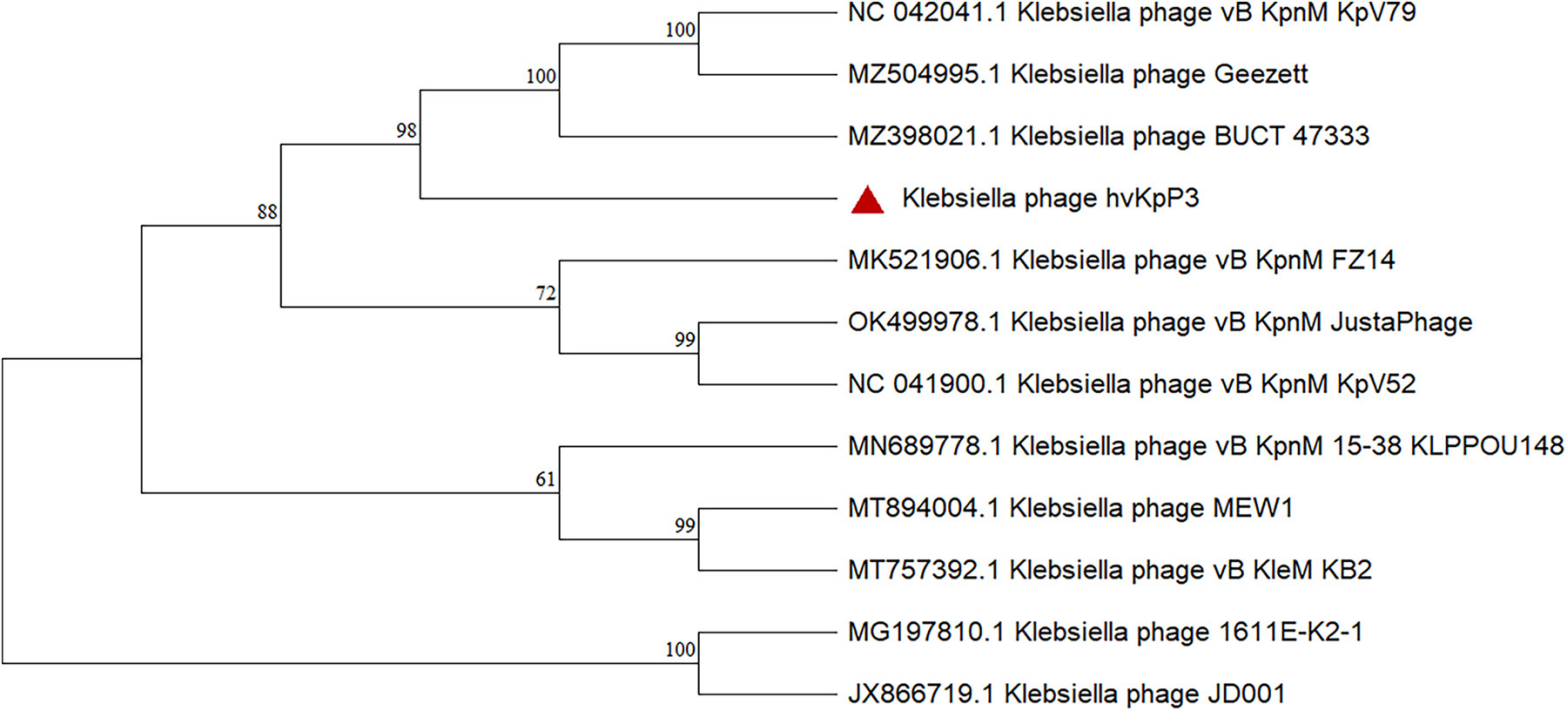
Pairwise BLASTn comparison and phylogenetic tree of hvKpP3 using JustaPhage. The genome map was created using Easyfig. Arrows represent predicted open reading frames; the direction of arrows represents the direction of transcription. Different colors denote different functional groups of bacteriophage genes.

The whole genome of phage hvKpP3 was sequenced, analyzed, and deposited in the GenBank database under the accession number MT559528. The hvKpP3 phage has a 48,861-bp DNA genome with a G+C content of 48.6%. In total, gene annotation of the phage genome showed that the hvKpP3 genome contained 80 coding domain sequences (CDSs). The putative functions of the phage proteins were predicted by bioinformatics analysis, which showed that 18 coding domain sequences were functionally annotated, while 62 CDSs were annotated as hypothetical proteins. All of the annotated CDSs can be further categorized into five modules: (i) DNA replication and regulation (DNA polymerase, HNH endonuclease, deoxyuridine 5′-triphosphate nucleotidohydrolase, and helicase), (ii) lysis (lysozyme), (iii) structural proteins, (iv) DNA packaging (head morphogenesis protein, major capsid protein, tape measure protein, baseplate protein, phage noncontractile tail fiber protein Gp17, tail fiber protein), and (v) other proteins. Studies have shown that DNA polymerase and helicase play important roles in phage replication (16). Nucleotide BLAST analysis showed that phage hvKpP3 exhibited high DNA similarity (76%) to the KpnM JustaPhage (GenBank accession no. OK499978.1). Therefore, we compared the annotations of the phage hvKpP3 and KpnM JustaPhage genomes using Easyfig software (Fig. 3). Both phages belong to the family *Myoviridae*, but to different subfamilies, with some differences in their structural protein modules, which could explain why phages with similar genomes can infect different clinical strains. To analyze phage evolutionary relationships, we constructed a phylogenetic tree for comparative analysis using Mega 11. The results showed that the phages hvKpP3, KpnM, KpV79, Geezett, and BUCT4733 are closely related (Fig. 4).

### Isolation and sequencing of phage-resistant *K. pneumoniae*.

Although phage hvKpP3 treatment in the G. mellonella model produced significant effects, phage-resistant K. pneumoniae colonies were still readily produced on bacterium-phage cocultured LB agar plates. The results of the phage resistance mutation assay showed that the rate of phage resistance mutations was 4.98 × 10^−4^. We randomly isolated two phage-resistant mutants, hvKpP3R and hvKpP3R15. To identify the genes responsible for bacterial resistance, the genomes of WT hvKpLS8 and the phage-resistant mutant hvKpP3R were sequenced and analyzed using the Illumina HiSeq platform. HvKpP3R acquired a T→G transversion mutation in the proposed glycosyltransferase gene (named *GT*), which is located at the same position as fragments 1,815,874 to 1,876,801 of the genome available from NCBI under accession no. CP052258.1. This mutation led to premature termination of *GT* translation, as shown in Fig. S2 and S3. The *GT* gene is located in an LPS synthesis gene cluster between UDP-galactopyranose mutase *rfbD* and glycosyltransferase family 1 protein. In addition, hvKpP3R15 sequencing analysis revealed a deletion of the G base at position 16 in the *wcaJ* gene.

### Phenotypic characterization of phage-resistant *K. pneumoniae*.

**FIG 5:**
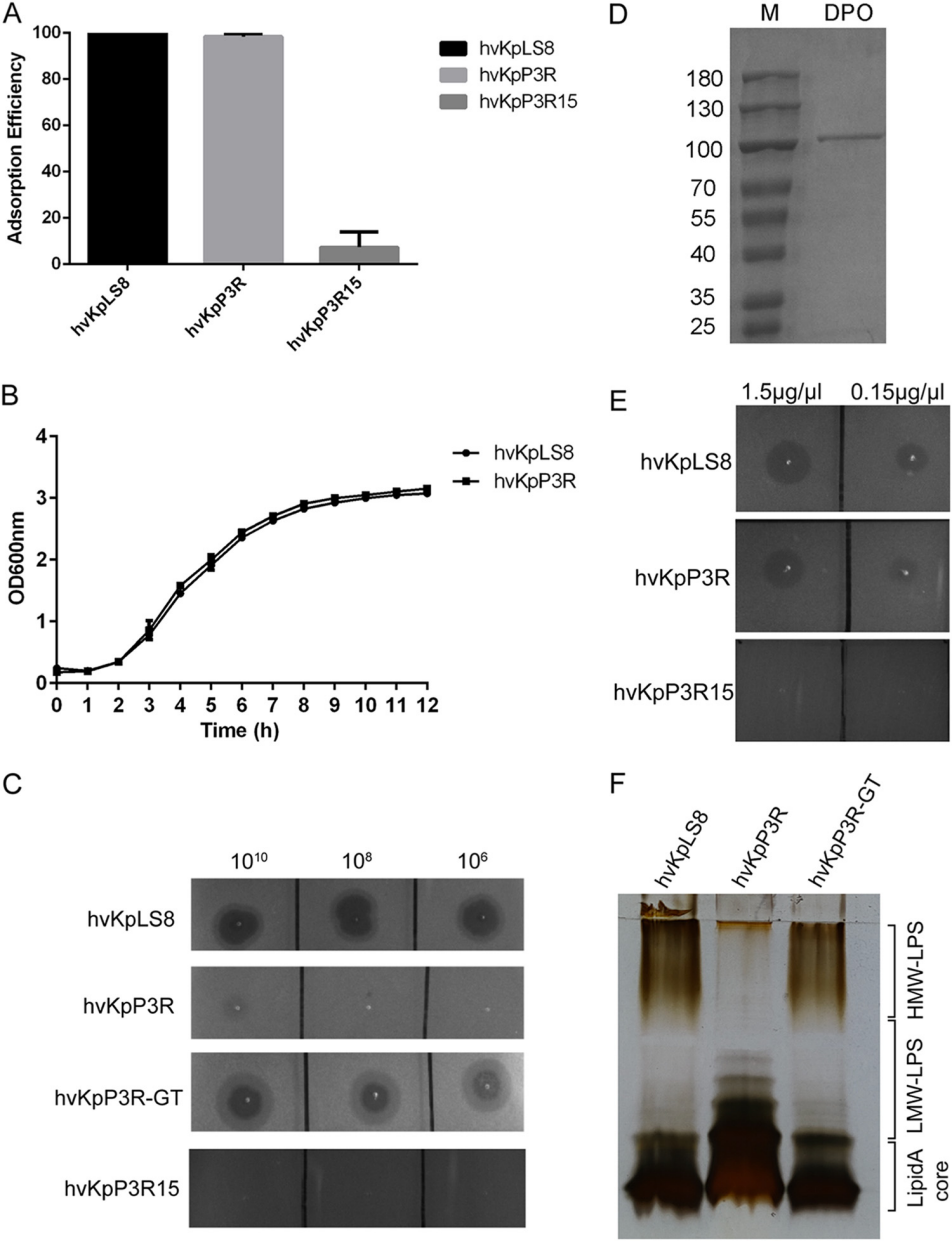
Characterization of phage-resistant K. pneumoniae. (A) Adsorption efficiencies of hvKpP3 binding to K. pneumoniae hvKpLS8, hvKpP3R, and hvKpP3R15. (B) Growth curves of the wild-type (WT) and hvKpP3R mutant K. pneumoniae strains. OD_600_, optical density at 600 nm. (C) K. pneumoniae WT hvKpLS8, mutant strains hvKpP3R15 and hvKpP3R, and complementation strain hvKpP3R-GT were grown to mid-log phase at 37°C and spread on lysogeny broth (LB) plates. Serial dilutions of phage hvKpP3 were dotted onto the bacterial lawn and grown at 37°C for 16 to 18 h. (D) Expressed glutathione *S*-transferase (GST)-tagged recombinant depolymerase (DPO) was purified on a glutathione-agarose column and then analyzed by SDS-PAGE in an 8% gel. “M” indicates the protein marker. (E) Confirmation of activity. Spot testing (1 μL) of the purified tail spike protein on a lawn of K. pneumoniae WT hvKpLS8 and mutants hvKpP3R and hvKpP3R15. (F) Lipopolysaccharide (LPS) specimens were prepared from K. pneumoniae hvKpLS8, mutant strain hvKpP3R, and complementation strain hvKpP3R-GT, which were separated by SDS-PAGE and visualized by silver staining.

Phage adsorption experiments were performed to explore the mechanism of bacteriophage resistance. The adsorption capacity of hvKpP3 to hvKpP3R15 decreased to 7.4% (Fig. 5A), similar to that of other *wcaJ* mutant strains in previous studies (17–19). However, the adsorption efficiency of the mutant strain, hvKpP3R, did not change. The growth of hvKpP3R cells was unaffected (Fig. 5B). It was found that a high concentration of phage did not produce plaque, but a halo on the hvKpP3R plate (Fig. 5C). Subsequently, we purified the recombinant depolymerase (DPO) protein to confirm the ability to degrade bacterial capsules of the host bacterium hvKpLS8 of the parent phage (Fig. 5D). Different concentrations of DPO protein were spotted on double-layer agar plates, and the recombinant DPO protein produced halos on plates of the resistant hvKpP3R bacteria, but not on the hvKpP3R15 plates (Fig. 5E). In addition, Alcian blue staining showed that hvKpP3R15 lacked high-molecular weight CPS compared to wild-type (WT) hvKpLS8. However, there was no change in hvKpP3R (Fig. S4). Therefore, these data indicate that the capsule is the receptor of phage hvKpP3 and is also a target of depolymerase. Meanwhile, it showed that the mutation of *GT* in hvKpP3R did not affect the capsule.

### Long-chain O-antigen is lost in the *GT* mutant strain.

**FIG 6:**
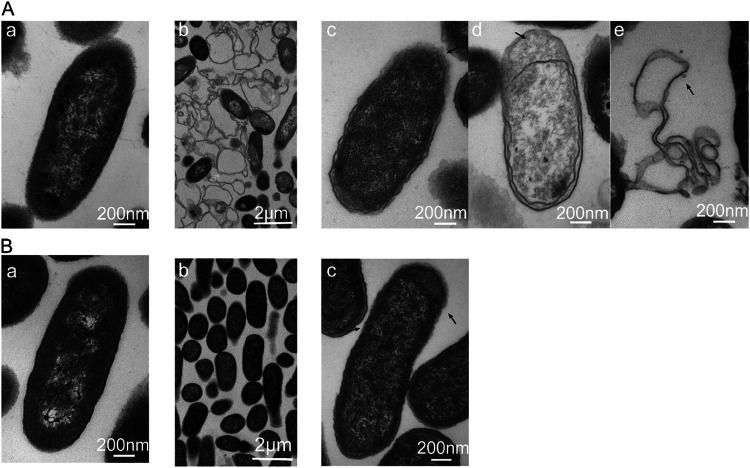
TEM images of WT strain hvKpLS8 and mutant hvKpP3R after 50 min incubation with phage hvKpP3. (A) Image a, WT strain hvKpLS8; image b, TEM images of WT strain hvKpLS8 after 50 min incubation with phage hvKpP3; image c, phages adsorb to the surface of strain hvKpLS8; image d, phage DNA enters the cell and replicates; image e, the cells lyse, releasing progeny phages. (B) Image a, the mutant strain hvKpP3R; image b, TEM images of mutant hvKpP3R after 50 min incubation with phage hvKpP3; image c, phages adsorb to the surface of strain hvKpPR.

To determine the consequences of *GT* modification, purified lipopolysaccharide (LPS) was detected by sodium dodecyl sulfate polyacrylamide gel electrophoresis (SDS-PAGE) of K. pneumoniae. Compared with the wild-type strain, no HMW-LPS was found, but more low-molecular weight LPS (LMW-LPS) was produced in the *GT* mutant as indicated by silver staining. In hvKpP3R-GT, fully restored HMW-LPS production was induced by introducing the pET28a-GT-expressing plasmid into the hvKpP3R mutant (Fig. 5F). At the same time, spot testing results showed that hvKpP3R-GT restored phage sensitivity with clear plaques (Fig. 5C). This suggests that the absence of HMW-LPS is a decisive factor in the failure of phages to infect the host. However, because the hvKpP3R strain was still capable of adsorbing phages, this suggests that the mechanism of resistance to lysis might be related to a certain process blocked after adsorption. To investigate this possibility, we observed ultrastructural changes in phage-infected bacteria by transmission electron microscopy using an ultrathin sectioning technique. After 50 min of incubation with phage hvKpP3, most of the wild-type strains of hvKpLS8 showed changes in cell membrane density, ruptured cell membranes, lysed cells, and successfully released phage particles (Fig. 6A). In contrast, phage capsids were adsorbed to the surface of the mutant strain hvKpP3R, but the next step of lysis could not be performed (Fig. 6B).

## DISCUSSION

In this study, we isolated a novel phage belonging to the family *Myoviridae*, hvKpP3, which exhibited high lytic activity against K2 hypervirulent K. pneumoniae. To date, *Myoviridae* phages have not been found to disrupt the hypervirulent K. pneumoniae MLST ST65 with a K2 serotype. Fibronectin is responsible for determining the host specificity of phages (1), which explains the difference in the serotypes of the host bacteria infected by *Myoviridae* phages isolated in this study compared with other reports (16, 20, 21). The phylogenetic tree showed that hvKpP3 is a new phage that is distinct from others. The hvKpP3 phage has a fast adsorption rate and a short proliferation time in the cells of host bacteria, and it can release a large number of phages in a short period of time. The hvKpP3 phage has good thermal and acid-base stability, which makes it a potential candidate for future clinical phage therapy. Genomic analysis shows that hvKpP3 contains no virulence, lysogenic, or antimicrobial resistance genes. These data indicate that hvKpP3 is suitable for phage therapy.

Phages undergo a complex series of processes during bacterial infection. However, bacteria have evolved a number of defense mechanisms and strategies to resist phage invasion. Using comparative genomics, we demonstrated for the first time that bacteria can acquire phage resistance through *GT* mutations. In our analysis of phage resistance mechanisms, we found that the cell attachment receptor of phage hvKpP3 is CPS. Deletion of *wcaJ* resulted in the disruption of capsular synthesis, which further significantly reduced phage adsorption capacity. Consistent with the results of Dunstan et al. (2), this finding suggests that the capsule plays a crucial role in phage adsorption. Notably, unlike in previous studies of K. pneumoniae phages with lipopolysaccharide as a receptor (22), LPS was not significantly related to adsorption in the present study.

Glycosyltransferase has been demonstrated to be essential for the biosynthesis of the O-antigen of bacterial lipopolysaccharides (23). Previous studies have shown that the downregulation of glycosyltransferase (GT-1, GT-2) expression is caused by epigenetic modifications, such as DNA methylation, which is related to capsular polysaccharide synthesis (17). Interestingly, in the present study, the phage-resistant strain hvKpP3R showed a loss-of-function mutation in the glycosyltransferase responsible for LPS biosynthesis. The production of incomplete LPS lacking HMW-LPS abolished phage plaque formation. In a study by Zhang et al. (24), mutations in glycosyltransferase resulted in disruption of the O-antigen region of LPS. The results of silver staining in our study clearly showed the absence of HMW-LPS. The changes in LPS profile indicate that the O-antigen is highly variable compared with the relatively conserved oligosaccharide nucleus (25). Glycosyltransferase polymerizes the completed O-antigen on a single Und-P carrier molecule in the inner leaflet of the internal membrane (IM) by utilizing nucleotide-activated sugar donors (26). Thus, deletion of the *GT* gene may result in the accumulation of LMW-LPS in cells and a reduction of HMW-LPS. To further validate the effect of *GT* mutation on phages, we restored phage susceptibility and lipopolysaccharide integrity by complementation experiments, which showed that HMW-LPS are the major limiting step in phage infection. Gonzalez-Garcia et al. (27) showed that contact of noncontractile-tail phages with LPS O-antigen receptors caused conformational changes in the tail apparatus and portal complex, leading to the opening of phage particles and the release of DNA content (28). Interestingly, we found that the tail fibronectin of hvKpP3 is also noncontractile, and transmission electron microscopy showed that the phage was halted at a certain stage after adsorption during the process of infecting the resistant bacteria hvKpP3R. We suspect two reasons for this phenomenon. On the one hand, the absence of HMW-LPS may cause the phage to lack the action site to trigger DNA ejection. However, it is also possible that the phage DNA could not enter the cytoplasm. In conclusion, our study demonstrated through gene sequencing and LPS silver staining that phage resistance is mainly linked to the loss of HMW-LPS, which is associated with *GT* mutation. However, the exact mechanism by which HMW-LPS loss causes phage resistance requires further investigation.

## MATERIALS AND METHODS

### Bacterial strains and growth conditions.

Eleven strains of K. pneumoniae were isolated from three hospitals in China (Table 1). The host bacteria used for phage isolation were provided by the Sixth Affiliated Hospital of Wenzhou Medical University and were named hvKpLS8. The remaining 11 strains were used to determine the range of infection of the isolated phages. All strains were identified by 16S rRNA gene PCR and stored at −80°C in 15% (vol/vol) glycerol (Table S1). All culturing was carried out in lysogeny broth (LB) containing 1% tryptone, 0.5% yeast extract, 1% NaCl, and 1.5% agar at 37°C with shaking at 200 rpm.

### Phage isolation and purification.

K. pneumoniae phages were isolated from sewage water collected from a wastewater treatment plant in Chengdu, Sichuan. Briefly, untreated sewage was mixed with a bacterial culture at a volume ratio of 1:1, then incubated at 37°C for 24 h in a shaker incubator with shaking at 200 rpm. The mixture was centrifuged (13,000 × *g*, 2 min), and the supernatants were filtered through a 0.22-μm pore membrane filter to remove bacterial debris. Next, the filtrate and bacteria were mixed and added to 4 to 6 mL of molten semisolid soft agar (0.7% agar), cooled to room temperature, poured over solidified 1.5% nutrient agar plates, and incubated at 37°C overnight. The resulting plaques were subjected to three rounds of plaque purification until uniform phage plaques formed. Purified phages were stored at 4°C in SM buffer (100 mM NaCl, 8 mM MgSO_4_ · 7H_2_O, and 50 mM Tris-HCl [pH 7.5]).

### Transmission electron microscopy of phages.

Phage particles were spotted onto a carbon-coated copper grid and negatively stained with 2% (wt/vol) phosphotungstic acid. After drying, phages were observed using a Tecnai G_2_ F20 electron microscope (FEI, Hillsboro, OR, USA) operating at 80 kV to acquire morphological information on single-phage particles. Phages were classified according to the International Committee on Taxonomy of Viruses (ICTV) guidelines (29).

### Assessment of hvKpP3 phage thermal and pH stability.

Thermal and acid-base stability tests were performed according to the methods of Jiang et al. (14), with some modifications. The phage solutions were diluted with SM buffer, after which the phages were treated at a specified temperature or pH. After incubation for 1 h, the titer of each phage sample was determined using the double-layer agar method.

### Adsorption experiments and one-step growth analysis.

To analyze the adsorption rate of the phages isolated in this study, phage suspensions were added to the culture of the exponential growth host strain at a multiplicity of infection (MOI) of 0.1 (18). Aliquots were retrieved at 1-min intervals over 10 min. The mixture was incubated at 37°C and centrifuged (13,000 × *g*, 1 min) immediately. The unadsorbed phage particles in the supernatant were enumerated using a double agar overlay plaque assay. Each experiment was performed in triplicate.

One-step growth assays were performed as described previously (30) with minor modifications, to determine the incubation period and outbreak volume of the phages. Phages were added to a fresh host culture at an MOI of 10. The mixture was placed in an incubator at 37°C for 10 min, then centrifuged at 12,000 × *g* for 1 min to remove the free phages. The pellet was then resuspended in 5 mL of fresh LB broth, and phage titers in the culture were determined at 10-min intervals. The experiments were conducted in triplicate.

### Host range determination and polysaccharide depolymerase experiments.

Spot tests were performed to observe phage infection as previously described (10). Twelve K. pneumoniae strains were used in phage lysis assays to determine the lytic host range of the phage. Briefly, the strains were grown overnight in LB medium. The phage or purified recombinant polysaccharide depolymerase of hvKpP3 expressed by BL21(DE3) (31) was spotted on LB agar bacterial lawn culture. Spots were recorded as (i) clear, phage can kill the bacteria; (ii) turbid, no lysis, but capsule depolymerase was active; and (iii) absent, the isolate was resistant to the phage.

The constructed plasmid was transformed into Escherichia coli BL21(DE3) cells and colonies were grown overnight at 37°C in LB medium supplemented with 50 mg/mL kanamycin. The recombinant isolates were cultured to the exponential growth phase, induced with 0.1 M isopropyl-β-d-thiogalactopyranoside (IPTG), and further grown for 16 h at 18°C. The cells were harvested by centrifugation (6,000 rpm, 4°C, 10 min), resuspended in PBS, then broken by ultrasound. The supernatant was collected, filtered, and loaded into a glutathione-agarose column. The bound protein was eluted with 25 mL solution A (20 mM Tris-HCl [pH 7.4], 0.2 mM EDTA, 0.5 mM phenylmethylsulfonyl fluoride, 1 M NaCl, 1 mM dithiothreitol) and 25 mL solution B (20 mM Tris-HCl [pH 7.4], 0.2 mM EDTA, 0.1 M NaCl). Pure protein fractions eluted with solution C (20 mM Tris-HCl [pH 7.4], 0.2 mM EDTA, 0.1 M NaCl, 15 mM glutathione) were collected. Protein purity was assessed by 8% (wt/vol) SDS-PAGE.

### Sequencing and analysis of bacteriophage genomes.

Phage DNA was extracted from the phage preparation as previously described (7), with some modifications. Briefly, to remove bacterial DNA and RNA, DNase and RNase were added to the phage preparation at a final concentration of 1 μg/mL, and the mixture was incubated overnight at 37°C. Lysis buffer (20 mM EDTA, 50 μg/mL proteinase K, and 0.5% [wt/vol] SDS in SM solution) was added to the purified phage stock solution. The mixture was incubated at 56°C for 1 h. Next, the sample was washed three times using an equal volume of a mixture comprised of phenol, chloroform, and isoamyl alcohol (25:24:1), followed by centrifugation at 4°C, 12,000 × *g* for 10 min to remove the debris. The supernatant was mixed with isoamyl alcohol and stored at −20°C for 1 h. The mixture was centrifuged at 4°C and 12,000 × *g* for 10 min, and the precipitated DNA was collected using sterile double-distilled water.

DNA samples were sequenced in the second generation using the Ion S5 genome sequencer (Thermo Fisher Scientific, Waltham, MA, USA) and in the third generation using the MinION (Oxford Nanopore Technologies, Oxford, United Kingdom) genome sequencer. Sequencing data were assembled using SPAdes assembler v3.13.2 (32), HGAP4, and Canu v1.6 MUMmer v3 (33) software was used to analyze the contigs by splicing the second- and third-generation sequencing data to reconfirm the assembly results, determine the positional relationship between the contigs, and determine the gaps between contigs. Genome annotations were checked through sequence comparisons of protein sequences using BLASTn software and Easyfig v2.2.3 to illustrate the genome map.

### Bacteriophage therapy assay.

Currently, G. mellonella is used as a model to evaluate the potential of phages against infection (34). Creamy white larvae weighing approximately 300 mg were selected for this assay. Larvae were starved for 24 h before the experiment, and both the bacterial and phage lysates were injected into the larvae through the last left proleg (35). In the *in vivo* experiment, the larvae were divided into four groups, with 20 randomly chosen larvae used for each group: (a) injected with 10 μL of PBS, (b) injected with 10 μL of 10^6^ CFU/mL host bacteria, (c) injected with 10 μL of 10^7^ PFU/mL of phage, and (d) injected with 10 μL of 10^6^ CFU/mL host bacteria and then with 10 μL of 10^7^ PFU/mL phage within 30 min. All larvae were incubated at 37°C, and dead larvae were counted at 12-h intervals up to 72 h after the incubation. All experiments were performed in triplicate and analyzed using GraphPad Prism v6.0.

### Screen for phage-resistant strains.

Phages were mixed with host bacteria for cultivation, and the mixtures were cultured on double-layer soft agar. Plates were incubated overnight at 37°C, and the resulting colonies were picked and saved for further assays.

### Bacterial growth curves and phage adsorption efficiency assay.

All strains were cultured as described above. The following day, cultures were incubated in LB at a concentration of 1 × 10^7^ CFU/mL and added to the individual wells of a 96-well microtiter plate. Plates were incubated for 12 h at 37°C, and absorbance readings at 600 nm were recorded every 30 min using a BMG SPECTROstar Nano Plate Reader (Imgen Technologies, Alexandria, VA, USA). Growth rates of the bacterial strains were calculated using three biological replicates.

Phage adsorption tests were performed on different K. pneumoniae strains according to a previously reported protocol (18). The host bacteria (1 × 10^9^ CFU/mL) and phage (10^6^ PFU) were mixed and incubated for 10 min at 37°C with shaking and then centrifuging. To determine the amount of unabsorbed phage, the phage titers remaining in the supernatant were evaluated based on an assay of the double-layer plates. The phage adsorption rate was calculated as ([initial titer – residual titer]/initial titer) × 100%. Three biological replicates were performed.

### Transmission electron microscopy of bacteria and ultrathin sectioning.

Transmission electron microscopy was performed using a JEM-1400PLUS microscope (JEOL, Tokyo, Japan). Bacterial samples were prepared as described previously with some modifications (36). Briefly, after coculture of phages and bacteria for 50 min, the precipitate was collected by centrifugation and washed three times with PBS. The samples were fixed for at least 2 h at room temperature in 3% glutaraldehyde, post-fixed with 1% osmium tetroxide, dehydrated in alcohol grades, incubated with propylene oxide, and infiltrated overnight with a 1:1 mixture of propylene oxide and low-viscosity epoxy resin. The following day, the samples were embedded in epoxy resin and polymerized. Ultrathin sections (approximately 50 nm) were cut on a Reichert EM UC7 microtome, transferred to copper grids stained with lead citrate, and examined and imaged using a JEM-1400PLUS TEM.

### Bacterial genome sequencing and analysis.

Genomic DNA of WT hvKpLS8 and phage-resistant mutants was sequenced at Sangon Biotech (Shanghai, China) using the Illumina HiSeq platform (~1 Gbp/sample, paired-end) as previously described. The quality of the raw sequencing reads was evaluated using FastQC software. Low-quality reads and adapter sequences were trimmed using Trimmomatic software. Following the Genome Analyzer Toolkit (GATK) best practices pipeline, we used the genomic mapping tool Burrows-Wheeler Aligner to map low-divergent sequences to the reference genome of K. pneumoniae. Mutations, including base substitutions, deletions, and insertions, were detected using SAMtools, MarkDuplicates, and BEDTools. The DNA deletion mutations were further validated using PCR and sequencing.

### Cloning and complementation.

Genomic DNA was used as the template for WT gene cloning via PCR. The primers used are listed in the supplemental material. The PCR products were purified and cloned into the pET-28a vector by homologous recombination using the ClonExpress II One Step Cloning kit (Vazyme, Nanjing, China). Recombinant plasmids were first heat-shocked into E. coli DH5α cells and further electroporated into the corresponding phage-resistant mutants. Complementation strains were verified by PCR and sequenced using pET28A primers (Table S1). Bacterial isolates transformed with an empty vector were tested in parallel.

### Extraction and analysis of LPS and CPS.

LPS were extracted from fresh overnight cultures of the wild-type, mutant, and complement strains as described previously (37), with some modifications. Dried bacterial cells (20 mg) were suspended in 10 mL of 30 mM Tris-HCl buffer (pH 8.1) and centrifuged (5,000 rpm, 15 min, 4°C). The precipitates were resuspended in 400 μL of solution containing 20% sucrose in 30 mM Tris-HCl (pH 8.1) and placed on ice. Then, 40 μL of lysozyme (1 mg/mL) in 100 mM EDTA (pH 7.3) was dissolved on ice for 30 min. Centrifuge tubes were placed at −80°C for 30 min. After repeated freeze-thaw cycles, 3 mM EDTA solution was added, shaken, and mixed well. To ensure complete cell breakage, the cell lysates were sonicated with 2-min bursts at a probe intensity of 75, after which less than 1% of the bacteria remained intact. Samples were centrifuged at 6,000 rpm for 15 min at 0 to 4°C. The supernatants were then centrifuged at 16,000 rpm for 1 h at 4°C. The pellets containing LPS were resuspended in distilled water. LPS preparations were applied to an SDS polyacrylamide gel containing 15% acrylamide and separated. Thereafter, LPS preparations were stained using the silver staining method.

For capsular polysaccharide analyses, K. pneumoniae strains were inoculated in LB medium and cultured overnight. Capsular polysaccharide of these strains was extracted with a CPS extraction kit (EX1750, Solarbio Life Sciences, Beijing) according to the manufacturer’s instructions. Samples were analyzed by 6% SDS-PAGE and subsequently stained with the cationic dye Alcian blue (38). Briefly, the gel was washed in fixing buffer (25% ethanol and 10% acetic acid in Milli-Q water) three times at 50°C (10 min each wash), before staining with 0.1% Alcian blue in fixing buffer (for 15 min at 50°C in the dark). The gel was destained with fixing buffer at room temperature and visualized.

### Statistical analysis.

All experiments were performed with *n* = 3 replications. Statistical analysis was performed using GraphPad Prism v6.0 (GraphPad Software Inc., La Jolla, CA, USA). For phage adsorption efficiency assays, comparisons between mutant and WT strains and between WT and complementation strains were evaluated for statistical significance using one-way analysis of variance. Survival was analyzed using Kaplan-Meier analysis with a log-rank test. Statistical significance was set at *P < *0.05.

### Data availability.

The phage hvKpP3 genome sequence data have been deposited in the GenBank database (https://www.ncbi.nlm.nih.gov/genbank/) under accession no. MT559528.
