# Supplementary material for: Resistance of Klebsiella pneumoniae to Phage hvKpP3 Due to High-Molecular Weight Lipopolysaccharide Synthesis Failure
Source: Microbiol Spectr. 2023 Apr 6;11(3):e04384-22. doi: 10.1128/spectrum.04384-22 (PMC10269817; doi:10.1128/spectrum.04384-22)
Supplement: Supplemental file 1 — Table S1 and Fig. S1 to S4. Download spectrum.04384-22-s0001.pdf, PDF file, 0.6 MB [file spectrum.04384-22-s0001.pdf]

Supplementary Table 1: Primers used in this study

gene	Primers Name	**Sequences (5’-3’)**
16S rRNA	F	AGAGTTTGATCCTGGCTCAG
	R	GGTTACCTTGTTACGACTT
GT	PET28A-*GT*-F	AGGAGATATACCATGG AATATACGGCATTGATAGTGAC
	PET28A-*GT*-R	TGGTGGTGGTGCTCGAG TTAATGATACTTACCACTAATACC
wcaJ	*wcaJ*-F	ATGACAATATCTCAGCATCGC
	wcaJ-R	GCAGCATCTTATCAATAAGCAG

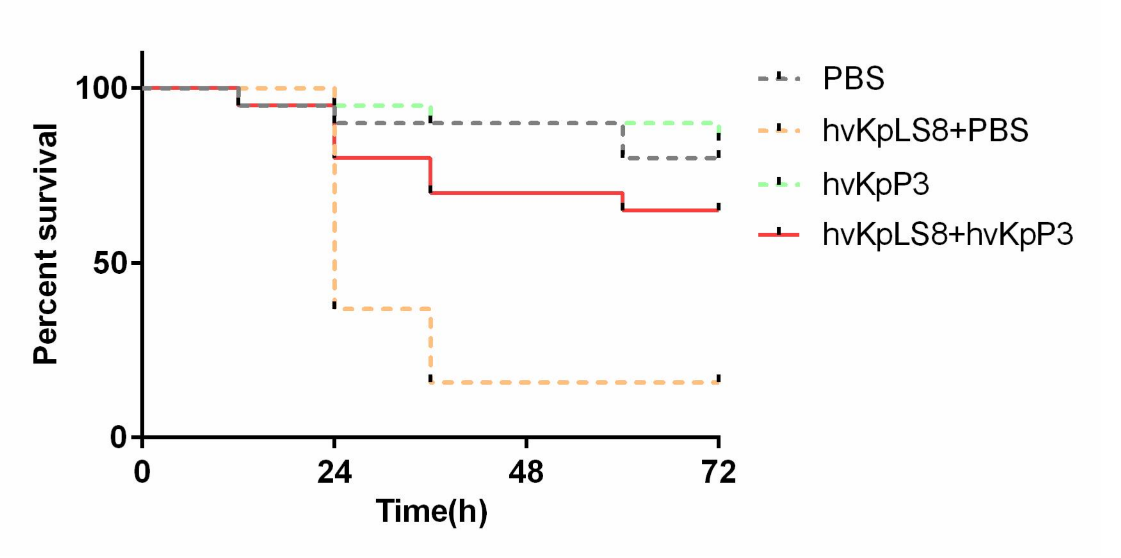

Supplementary Figure 1. Survival of *G. mellonella* larvae after infection by hvKpLS8 and phage therapy. Treatment with hvKpP3.

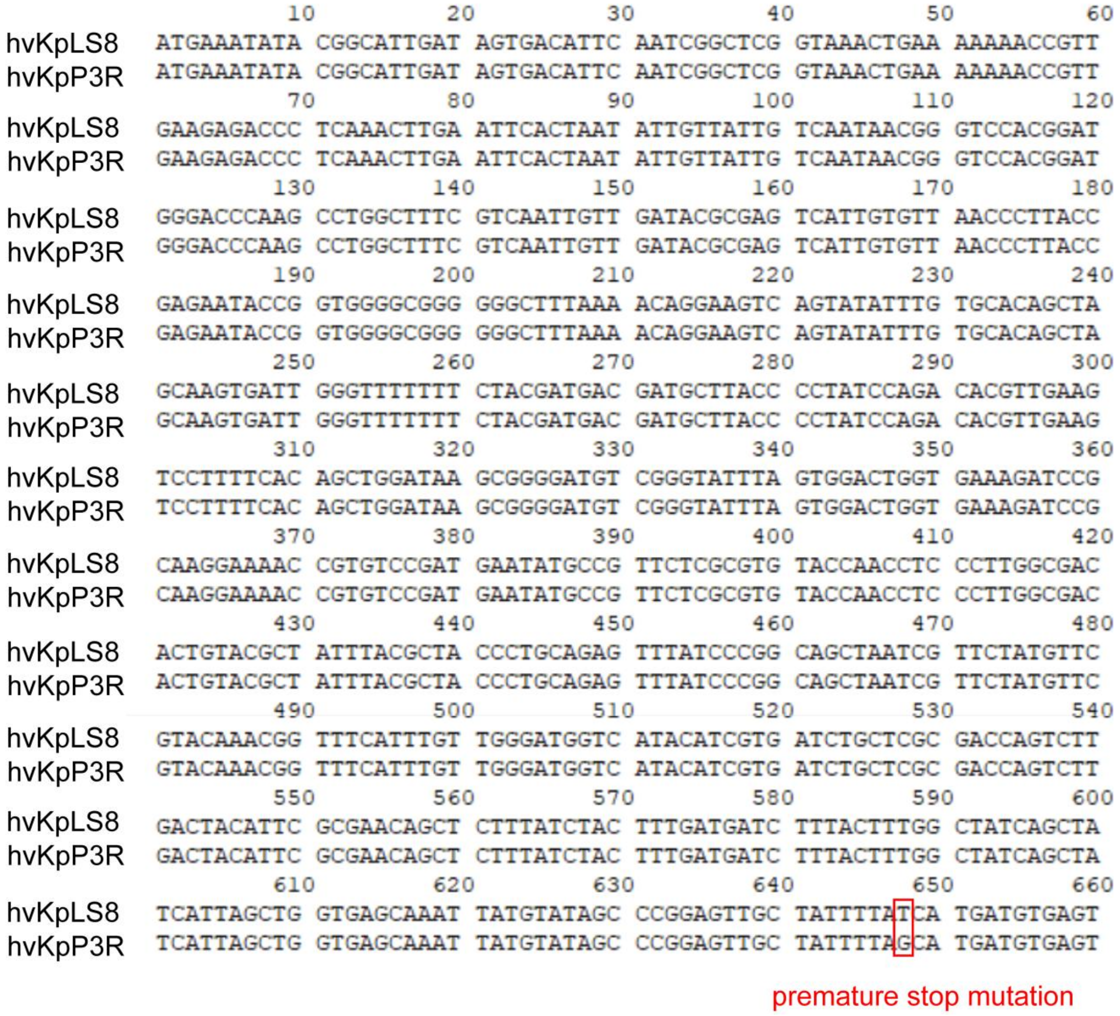

Supplementary Figure 2: Information about the mutation site of phage-resistant mutant hvKpP3R.

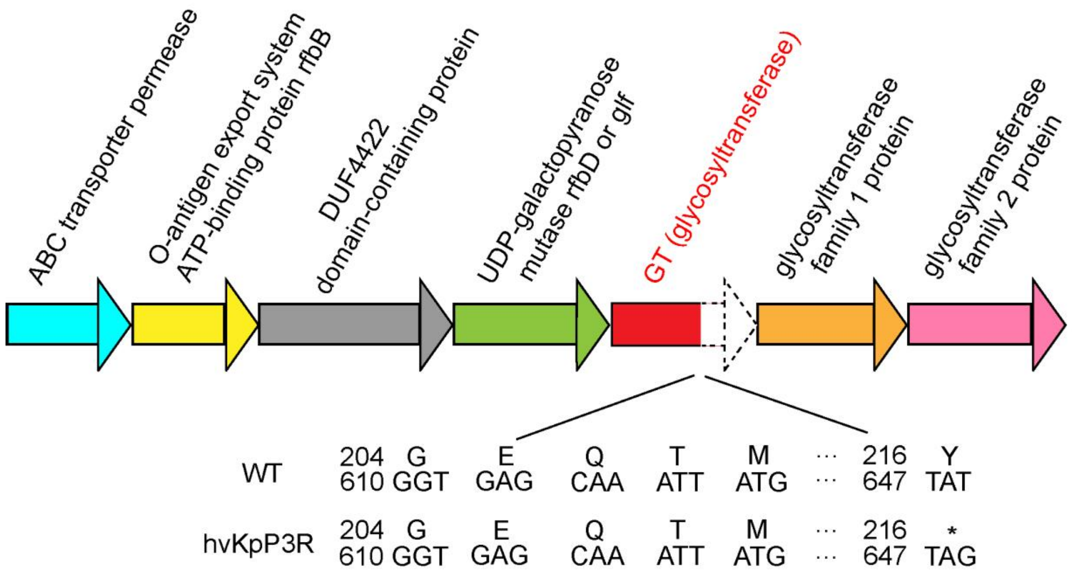

Supplementary Figure 3: Annotation of protein.

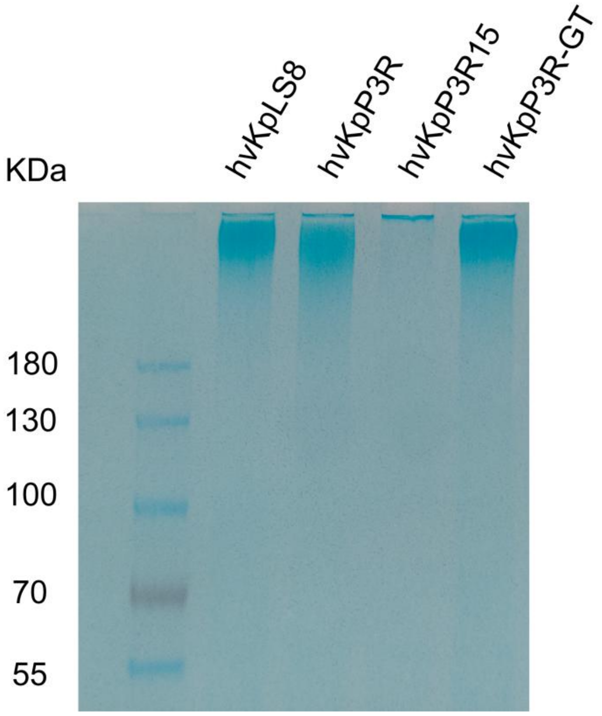

Supplementary Figure 4： CPS samples were extracted from *K. pneumoniae* strains. After separated by 6% SDS-PAGE, CPS phenotypes of hvKpLS8, hvKpP3R, hvKpP3R15 and hvKpP3R-GT were visualized by Alcian blue staining.
